# Prevalence of Obesity and Its Influence on Achievement of Cardiometabolic Therapeutic Goals in Chinese Type 2 Diabetes Patients: An Analysis of the Nationwide, Cross-Sectional 3B Study

**DOI:** 10.1371/journal.pone.0144179

**Published:** 2016-01-04

**Authors:** Xianghai Zhou, Linong Ji, Xingwu Ran, Benli Su, Qiuhe Ji, Changyu Pan, Jianping Weng, Changsheng Ma, Chuanming Hao, Danyi Zhang, Dayi Hu, CCMR Advisory Board and CCMR-3B Study Investigators

**Affiliations:** 1 Department of Endocrinology and Metabolism, Peking University People’s Hospital, Beijing, China; 2 Peking University Diabetes Center, Beijing, China; 3 Department of Endocrinology & Metabolism, West China Hospital, Sichuan University, Chengdu, China; 4 Department of Endocrinology & Metabolism, The Second Affiliated Hospital of Dalian Medical University, Dalian, China; 5 Department of Endocrinology, Xijing Hospital, Forth Military Medical University, Xi’an, China; 6 Department of Endocrinology, Chinese PLA General Hospital, Beijing, China; 7 Department of Endocrinology and Metabolism, The Third Affiliated Hospital Sun Yat-sen University, Guangzhou, China; 8 Department of Cardiology, Beijing Anzhen Hospital, Capital Medical University and Beijing Institute of Heart Lung and Blood Vessel Diseases, Beijing, China; 9 Division of Nephrology, Huashan Hospital, Fudan University, Shanghai, China; 10 Vital Strategic Research Institute, Berwyn, Pennsylvania, United States of America; 11 Department of Cardiology, Peking University People’s Hospital, Beijing, China; Children's National Medical Center, Washington, UNITED STATES

## Abstract

**Background:**

There are few data on the prevalence of obesity and its influence on achieving blood glucose, blood pressure, and blood lipid (3B) goals in Chinese type 2 diabetes outpatients.

**Methods:**

Patient demographic data, anthropometric measurements, medications, and blood glucose and lipid profiles of 24,512 type 2 diabetes patients from a large, geographically diverse study (CCMR-3B) were analyzed. Using cut-points for body mass index (BMI) and waist circumference (WC) recommended by the Working Group on Obesity in China, overweight and obesity were defined as BMIs of 24–27.9kg/m^2^ and ≥28.0kg/m^2^. Central obesity was defined as a waist circumference ≥80cm in women and ≥85cm in men. The 3B therapeutic goals were HbA1c<7.0%, BP<140/90mmHg and LDL-C<2.6mmol/L.

**Results:**

Overall, 43.0% of type 2 diabetes patients were overweight and 16.7% were obese; 13.3% of overweight and and10.1% of obese patients achieved all the 3B target goals. Overweight or obese patients were less likely to achieve 3B goals than those with normal BMIs. More than a half the overweight or obese patients (69.6%) were centrally obese. Patients with abdominal obesity were less likely to achieve cardiometabolic targets than those without abdominal obesity. In multivariate logistic regression analysis, female, higher BMI and waist circumference, smoking, drinking, sedentary lifestyle, and longer diabetes duration were significantly correlated with failure to achieve 3B control goals.

**Conclusions:**

Obesity is highly prevalent and associated with poor 3B control in Chinese type 2 diabetes patients. In clinical practice, more attention and resources should focus on weight loss for such patients.

## Introduction

The global prevalence of diabetes is rapidly increasing, and in China, the estimated prevalence of type 2 diabetes in adults rose from 2.5% in 1994 to 9.7% in 2007–2008 [1, 2]. There is strong evidence from epidemiological studies showing that obese diabetes patients are at increased risk of cardiometabolic diseases including hyperglycemia, hypertension, and dyslipidemia [3–6]. A meta-analysis of five longitudinal cohort studies demonstrated that overweight or obese diabetes patients had a twofold greater relative risk of mortality than normal-weight patients[7]. However, currently no studies have directly compared the rates of achieving therapeutic goals for control of hyperglycemia, hypertension, and dyslipidemia in normal weight and obese patients with diabetes who had been receiving medical care in hospital setting.

Cardiovascular events contribute to morbidity and mortality from diabetes, but control of cardiometabolic risk factors among diabetes patients is reported to be poor [8]. Identification of the factors that contribute to poor control would help to reduce morbidity and mortality in these patients. Overweight and obesity are characterized by excessive body fat accumulation that impairs both physical and psychosocial health. General obesity, i.e., overall body fat, is usually estimated by the body mass index (BMI), and waist circumference (WC), which measures abdominal fat, is the easiest way to assess central obesity. A survey of 233 type 2 diabetes patients, found that control of hyperglycemia, hypertension, and dyslipidemia was poor in 65.7%, 51.9%, and 97.1% of the subjects respectively, and that 60.1% of them were either overweight or obese [8]. Study supports the combined use of general and central obesity, as measured by BMI and WC, to assess risk of cardiovascular diseases in patients with diabetes and metabolic disorders [9].

Current type 2 diabetes treatment guidelines recommend multifactorial intervention, including both lifestyle modification and pharmacologic treatment [10,11]. However, even with multifactorial interventions, it is not known if diabetes patients with different BMIs have similar success in achieving recommended target goals for control of hyperglycemia, hypertension, and dyslipidemia in a real-life setting. We evaluated a population of overweight and obese patients from the Nationwide Assessment of Cardiovascular Risk Factors: Blood Glucose, Blood Pressure, and Blood Lipid (3B) study, a large, cross-sectional investigation of Chinese patients with type 2 diabetes [12]. We aimed to assess the achievement of 3B goals in this population and to compare the proportions of patients with different BMIs who achieved the target therapeutic goals set by the parent study. The influence of lifestyle on achieving the 3B targeted goals was assessed.

## Materials and Methods

### Study design and participants

The 3B study was conducted from August 2010 to March 2011 in endocrinology, cardiology, nephrology, and internal medicine clinics in 104 hospitals across all major geographic regions in China (registered in clinicaltrials.gov, NCT01128205). The study design and population have been described previously [12]. Briefly, it was a purely observational study, as only available data were documented, and patient treatment or assessment was not changed by the study. Endocrinology, cardiology, nephrology, and internal medicine specialists conducted the study. Consecutive outpatients were eligible for inclusion if they were 18 years of age or older and were diagnosed with type 2 diabetes at least 6 months prior to study screening. The study protocol was approved by the Ethics Committees of Peking University People’s Hospital, China–Japan Friendship Hospital, The Third Affiliated Hospital of Sun Yat-sen University, Huashan Hospital Fudan University, Shanghai Changzhen Hospital, Jiangsu Province Hospital, and The Second Affiliated Hospital of Dalian Medical University. All patients provided written informed consent to participate. Of 25,454 eligible patients, 610 with a BMI <18.5 kg/m^2^, and 23 with missing data, including BMI, hemoglobin A1c (HbA1c), total cholesterol (TC), low density lipoprotein cholesterol (LDL-C), high density lipoprotein cholesterol (HDL-C), or triglycerides (TG), were excluded. An additional 309 participants with extreme values (i.e., below the 0.1 percentile or above the 99.9 percentile) of age, BMI, duration of diabetes, LDL-C, TC, HDL-C, TG, and HbA1c were excluded. The remaining 24,512 patients (11,543 men and 12,969 women) were included in this study.

### Data collection

Patient data were collected from medical charts and clinical examinations conducted at one outpatient visit. Information on health behavior (i.e., smoking status, alcohol consumption, and exercise intensity), current medications, and personal history of hypertension and major cardiovascular diseases were obtained by self-reporting during a face-to-face interview. Physical examination included anthropometry and blood pressure measurements; height and weight were measured with patients standing with bare feet and light clothing on a mechanical balance. Fasting serum glucose, TC, LDL-C, HDL-C, and TG were measured at each study site with an automated analyzer. An HbA1c concentration known to have been obtained during the 3 months prior to the enrollment visit, or obtained at enrollment, was recorded.

### Definitions of variables

Smoking was defined as consuming an average of at least one cigarette daily for at least 1 year. Drinking was defined as consuming an average at least 50 g of alcohol daily for at least 1 year. A sedentary lifestyle was defined as not participating in regular physical activities.

BMI was calculated as weight in kilograms divided by the square of height in meters (kg/m^2^). As recommended by the Working Group on Obesity in China, normal weight was defined as a BMI of 18.5–23.9 kg/m^2^, overweight as a BMI of 24.0–27.9, and obesity as a BMI of 28.0 kg/m^2^ or higher [13–15]. A normal WC was defined as <80cm in women and <85 cm in men; WCs ≥80cm in women and ≥85cm in men indicated central obesity [13].

Each patient was evaluated for attainment of 3B study therapeutic control goals. According to the China Guidelines for Type 2 Diabetes (2013) and the American Diabetes Association recommendations (2015) [16,17], attainment of the glycemic goal was defined as an HbA1c <7%, the blood pressure (BP) goal was a systolic blood pressure (SBP) <140mmHg and a diastolic blood pressure (DBP) <90mmHg regardless of a history of hypertension or current antihypertensive treatment. The LDL-C goal was <2.6mmol/l regardless of a history of dyslipidemia or current anti-hyperlipidemia treatment.

### Statistical analysis

Statistical analysis was performed using SAS version 9.2 (SAS Institute Inc., Cary, NC, USA). Quantitative variables were expressed as means±standard deviation (SD) or medians and percentiles (25^th^ percentile, 75^th^ percentile). Categorical variables were reported as numbers and percentages. Between-group differences of continuous variables were tested for significance using one-way analysis of variance (ANOVA), and the chi-square or Fisher’s exact test were used for categorical data. Logistic regression analysis was used to determine the independent association between attainment of 3B therapeutic goals and the variables of age, gender, smoking status, alcohol consumption, sedentary lifestyle, BMI, WC, diabetes duration, and diabetes complications. Backward elimination (= 0.05) was performed. The results were expressed as odds ratios (ORs) with 95% confidence intervals (CIs). Statistical significance was accepted as a two-sided test with an alpha level of 0.05.

## Results

### Demographic and clinical characteristics

The demographic and clinical characteristics of the 24,512 patients with type 2 diabetes were stratified by BMI and WC and are shown in Table 1. The prevalence of overweight was 43.0% (*n* = 10,548), and 16.7% (*n* = 4098) of the patients were obese; 11,543 (47.1%) were men and 12,969 (52.9%) were women. The mean age of normal-weight diabetes patients was 63.5±11.6years, overweight patients were 62.2±11.7years of age, and obese patients were 60.8±12.4years of age (*P*<0.001). Obese patients had a shorter diabetes duration than normal-weight patients, but were more likely to have comorbidities (hypertension or hypertension with dyslipidemia). Alcohol consumption and sedentary lifestyle were more common in obese than in normal-weight patients. A greater percentage of overweight and obese patients had diabetes complications, including cardiovascular diseases, cerebrovascular disease, and nephropathy, compared with normal-weight patients (all *P*<0.05).

**Table 1:** Demographic and clinical characteristics of diabetes patients stratified by BMI and WC.

	Total(*n* = 24,512)	BMI (kg/m^2^)				BMI ≥24kg/m^2^		
		<24(*n* = 9866)	24–27.9(*n* = 10,548)	≥28(*n* = 4098)	*P*	Normal WC(*n* = 2904)	Central obesity(*n* = 6642)	*P*
Age, years (mean±SD)	62.5±11.8	63.5±11.6	62.2±11.7	60.8±12.4	<0.001	62.0±11.8	60.6±12.1	<0.001
<45	1697 (6.9)	576 (5.8)	717 (6.8)	404 (9.9)	<0.001	202 (7.0)	631 (9.5)	<0.001
45–54	4408 (18.0)	1615 (16.4)	1968 (18.7)	825 (20.1)		572 (19.7)	1386 (20.9)	
55–64	7323 (29.9)	2856 (28.9)	3263 (30.9)	1204 (29.4)		888 (30.6)	2032 (30.6)	
≥65	11084 (45.2)	4819 (48.8)	4600 (43.6)	1665 (40.6)		1242 (42.8)	2593 (39.0)	
Male	11543 (47.1)	4524 (45.9)	5291 (50.2)	1728 (42.2)	<0.001	1345 (46.3)	4614 (69.5)	<0.001
Diabetes duration, years, median (25^th^, 75^th^ percentile)	6.2 (2.7, 11.3)	6.7 (2.7, 12.0)	6.0 (2.7,11.0)	5.9 (2.5, 10.9)	<0.001	6.0 (2.6, 11.0)	5.7 (2.4, 10.8)	0.001
<1	2210 (9.0)	897 (9.1)	950 (9.0)	363 (8.9)	<0.001	265 (9.1)	653 (9.8)	0.002
1–4	8135 (33.2)	3101 (31.4)	3578 (33.9)	1456 (35.5)		967 (33.3)	2399 (36.12)	
5–9	5878 (24.0)	2289 (23.2)	2579 (24.5)	1010 (24.6)		704 (24.2)	1622 (24.42)	
≥10	8289 (33.8)	3579 (36.3)	3441 (32.6)	1269 (31.0)		968 (33.3)	1968 (29.6)	
Comorbidities*	17756 (72.4)	6447 (65.3)	7931 (75.2)	3378 (82.4)	<0.001	2084 (71.8)	5079 (76.5)	<0.001
Hypertension	7367 (30.1)	2926 (29.7)	3150 (29.9)	1291 (31.5)		866 (29.8)	1942 (29.2)	
Dyslipidemia	3018 (12.3)	1156 (11.7)	1404 (13.3)	458 (11.2)		351 (12.1)	973 (14.7)	
Hypertension and Dyslipidemia	7371 (30.1)	2365 (24.0)	3377 (32.0)	1629 (39.8)		867 (29.9)	2164 (32.6)	
Smoking	4052 (16.5)	1433 (14.5)	1921 (18.2)	698 (17.0)	<0.001	471 (16.2)	1669 (25.1)	<0.001
Alcohol consumption	1959 (8.0)	607 (6.2)	958 (9.1)	394 (9.6)	<0.001	227 (7.8)	876 (13.2)	<0.001
Sedentary lifestyle	8824 (36.0)	3403 (34.5)	3692 (35.0)	1729 (42.2)	<0.001	1031 (35.5)	2313 (34.8)	0.522
Diabetes complications								
Cardiovascular diseases	3653 (14.9)	1266 (12.8)	1663 (15.8)	724 (17.7)	<0.001	458 (15.8)	1004 (15.1)	0.413
Cerebrovascular disease	2469 (10.1)	981 (9.9)	1061 (10.1)	427 (10.4)	0.694	245 (8.4)	653 (9.8)	0.032
Peripheral vascular diseases	370 (1.5)	161 (1.6)	132 (1.3)	77 (1.9)	0.009	31 (1.1)	85 (1.3)	0.384
Nephropathy	3525 (14.4)	1271 (12.9)	1540 (14.6)	714 (17.4)	<0.001	377 (13.0)	1021 (15.4)	0.002
Retinopathy	4018 (16.4)	1698 (17.2)	1645 (15.6)	675 (16.5)	0.008	409 (14.1)	891 (13.4)	0.380
Neuropathy	3702 (15.1)	1486 (15.1)	1545 (14.6)	671 (16.4)	0.032	387 (13.3)	933 (14.1)	0.348

Data are expressed as *n* (%) unless otherwise indicated.

* Comorbidities included hypertension only, dyslipidemia only, and both conditions.

Of the 9546 overweight patients with a BMI ≥24kg/m^2^ (excluding 5,100 patients with missing WC data), 6642 (69.6%) were centrally obese; and of those, 69.5% were men. A significantly larger percentage of patients with central obesity had comorbidities compared with those who had a normal WC (76.5% *vs*. 71.8%, *P*<0.001). Centrally obese patients were also more likely to be smokers or alcohol drinkers (*P*<0.001).

### Medication regimens

The numbers and percentages of patients receiving pharmaceutical treatment for diabetes, hypertension, and dyslipidemia are shown in Table 2. The most frequently reported oral antidiabetic agents were metformin (38.6%), followed by sulfonylureas (28.9%) and -glucosidase inhibitors (26.6%). Use of antihypertensive, lipid lowering and oral antidiabetic agents was most frequent in obese patients. More than half of the obese patients (52.3%) were taking antihypertensive agents, and more than a quarter (27.9%) were taking blood-lipid lowering agents, compared with 37.6% and 19.8% of normal-weight patients, respectively (*P*<0.001). More centrally obese patients than patients with a normal WC were taking antihypertensive and oral antidiabetic agents (*P*<0.001). Interestingly, insulin treatment did not vary among patients with different BMI.

**Table 2:** Medication regimens of diabetes patients stratified by BMI and WC.

		BMI (kg/m^2^)				BMI ≥24kg/m^2^		
	Total(*n* = 24,512)	<24(n = 9866)	24–27.9(n = 10,548)	≥28(n = 4098)	*P*	Normal WC(*n* = 2904)	Central obesity(*n* = 6642)	*P*
Antihypertensive agents	10577 (43.2)	3711 (37.6)	4722 (44.8)	2144 (52.3)	<0.001	1244 (42.8)	2965 (44.6)	<0.001
Lipid lowering agents	5776 (23.6)	1954 (19.8)	2679 (25.4)	1143 (27.9)	<0.001	715 (24.6)	1727 (26.0)	0.473
Oral anti-diabetic agents	17992 (73.4)	7140 (72.4)	7753 (73.5)	3099 (75.6)	<0.001	2019 (69.5)	4919 (74.1)	<0.001
Metformin	9460 (38.6)	3314 (33.6)	4216 (40.0)	1930 (47.1)	<0.001	1064 (36.6)	2774 (41.8)	0.131
α-glucosidase inhibitors	6527 (26.6)	2664 (27.0)	2774 (26.3)	1089 (26.6)	0.031	696 (24.0)	1735 (261.)	0.424
Sulfonylureas	7075 (28.9)	3038 (30.8)	3016 (28.6)	1021 (24.9)	<0.001	752 (25.9)	1816 (27.3)	0.664
Thiazolidinediones	1820 (7.4)	677 (6.9)	782 (7.4)	361 (8.8)	0.289	159 (5.5)	530 (8.0)	0.098
Insulin	8688 (35.4)	3414 (34.6)	3788 (35.9)	1486 (36.3)	0.072	1077 (37.1)	2334 (35.1)	0.068

Data are expressed as *n* (%)

### Control of blood glucose, blood pressure and blood lipids

The 3B profiles and goal-attainment rates of the study patients are shown in Table 3. Overweight and obese patients had higher SBP, DBP, and fasting serum glucose, HbA1c, TC, LDL-C and TG, but lower HDL-C levels than normal-weight patients (*P*<0.05). Centrally obese patients had higher HbA1c and TG levels, but lower HDL-C levels than did patients with a normal WC (*P*<0.05).

**Table 3:** 3B profiles and goal attainment rates of diabetes patients stratified by BMI and WC.

		BMI (kg/m^2^)				BMI ≥24 kg/m^2^		
	Total(*n* = 24,512)	<24(n = 9866)	24–27.9(n = 10,548)	≥28(n = 4098)	*P*	Normal WC(*n* = 2904)	Central obesity(*n* = 6642)	*P*
SBP (mmHg)	133.1±15.7	131.2±15.4	133.8±15.5	135.9±16.1	<0.001	133.4±15.5	133.5±15.4	0.797
DBP (mmHg)	78.9±8.9	77.3±8.5	79.4±8.8	81.4±9.5	<0.001	79.7±9.0	80.0±9.0	0.088
FPG (mmol/L)	8.4±3.4	8.3±3.5	8.4±3.3	8.6±3.3	<0.001	8.4±3.5	8.4±3.2	0.849
HbA1c (%)	7.6±2.0	7.5±2.1	7.6±1.9	7.8±1.9	<0.001	7.5±2.0	7.7±1.9	0.003
TC (mmol/L)	4.97±1.21	4.95±1.22	4.97±1.20	5.01±1.21	0.015	5.01±1.24	4.92±1.18	0.092
LDL-C (mmol/L)	2.84±0.91	2.82±0.90	2.95±0.91	2.86±0.92	0.014	2.84±0.92	2.83±0.90	0.570
HDL-C (mmol/L)	1.30±0.43	1.35±0.44	1.27±0.42	1.26±0.44	<0.001	1.29±0.45	1.24±0.41	<0.001
TG (mmol/L)	1.98±1.65	1.76±1.42	2.09±1.75	2.25±1.80	<0.001	2.03±1.49	2.17±1.93	<0.001
HbA1c <7.0%	11,000 (44.9)	4793 (48.6)	4623 (43.8)	1584 (38.7)	<0.001	1341 (46.2)	2834 (42.7)	0.001
BP <140/90 mmHg	9885 (40.3)	4655 (47.2)	4000 (37.9)	1230 (30.0)	<0.001	1930 (66.5)	4313 (64.9)	0.150
LDL-C <2.6 mmol/L	10,426 (42.5)	4287 (43.5)	4427 (42.0)	1712 (41.8)	0.057	1254 (43.2)	2858 (43.0)	0.890
3B at goal	3512 (14.3)	1696 (17.2)	1401 (13.3)	415 (10.1)	<0.001	426 (14.7)	853 (12.8)	0.016

FPG, fasting plasma glucose. *3B goal was BP<140/90mmHg, LDL-C<2.6mmol/L, and HbA1c<7.0%. Data are shown as means±SD or *n* (%).

A total of 44.9% of the patients reached the recommended glycemic control target (HbA1c <7%); 40.3% achieved the BP target (<140/90mmHg), and 42.5% reached the lipid control target (LDL-C <2.6mmol/L). All 3B target goals (i.e., control of HbA1c, BP, and LDL-C) were achieved by 14.3% of the study participants. A total of 48.6% of normal-weight patients achieved HbA1c control, 47.2% achieved BP control, and 17.2% achieved all the 3B target goals compared with 38.7%, 30.0% and 10.1% of obese patients, respectively (*P*<0.001). A similar trend was seen in centrally obese patients, suggesting that patients who had an abnormal BMI and an abnormal WC were at increased risk of failure to achieve target therapeutic goals.

### Risk factors that influence the 3B control

Logistic regression analysis showed (Table 4) that higher BMI (OR = 1.26; 95%CI, 1.15–1.38 for BMI = 24–27.9kg/m^2^ and OR = 1.62; 95%CI, 1.37–1.93 for BMI ≥28 kg/m^2^), higher WC (OR = 1.01; 95%CI, 1.01–1.02), smoking (OR = 1.38; 95%CI, 1.22–1.57), drinking (OR = 1.31; 95%CI, 1.10–1.55), and longer diabetic duration (OR = 1.69; 95%CI, 1.46–1.95 for 5–9 years and OR = 1.45; 95% CI, 1.25–1.68 for ≥10 years) made it less likely to achieve 3B control goals. Male patients (OR = 0.78; 95%CI, 0.71–0.86) and frequent exercisers (OR = 0.85; 95%CI, 0.78–0.93) were more likely to achieve all 3B control targets.

**Table 4:** Factors associated with failure to achieve 3B goals by logistic regression analysis.

Variables	BP <140/90mmHg		LDL-C <2.6mmol/L		HbA1c <7.0%		3B at goal	
	OR (95% CI)	*P*	OR (95% CI)	*P*	OR (95% CI)	*P*	OR (95% CI)	*P*
Age (years), vs. <45years								
45–54	1.63 (1.39, 1.91)	<0.001	1.02 (0.90, 1.16)	0.727	0.76 (0.66, 0.87)	<0.001	1.05 (0.88, 1.25)	0.611
55–64	1.89 (1.62, 2.20)	<0.001	1.15 (1.02, 1.31)	0.022	0.56 (0.50, 0.64)	<0.001	1.03 (0.87, 1.23)	0.699
≥65	2.29 (1.96, 2.66)	<0.001	0.94 (0.83, 1.07)	0.340	0.48 (0.43, 0.55)	<0.001	0.85 (0.72, 1.01)	0.060
Gender (male vs. female)	0.98 (0.91, 1.06)	0.616	0.67 (0.62, 0.72)	<0.001	0.97 (0.90, 1.04)	0.350	0.78 (0.71, 0.86)	<0.001
Smoking (yes vs. no)	1.00 (0.91, 1.10)	0.991	1.12 (1.02, 1.22)	0.012	1.41 (1.29, 1.54)	<0.001	1.38 (1.22, 1.57)	<0.001
Drinking (yes vs. no)	1.18 (1.05, 1.33)	0.006	1.11 (0.99, 1.24)	0.067	1.15 (1.03, 1.29)	0.016	1.31 (1.10, 1.55)	0.002
Exercise (yes vs. no)	0.87 (0.82, 0.94)	<0.001	0.99 (0.93, 1.04)	0.653	0.77 (0.72, 0.82)	<0.001	0.85 (0.78, 0.93)	<0.001
BMI (kg/m^2^) vs. <24kg/m^2^								
24–27.9	1.30 (1.21, 1.40)	<0.001	1.04 (0.97, 1.11)	0.298	1.11 (1.04, 1.19)	0.002	1.26 (1.15, 1.38)	<0.001
≥28	1.66 (1.48, 1.87)	<0.001	1.03 (0.92, 1.16)	0.567	1.25 (1.11, 1.4)	<0.001	1.62 (1.37, 1.93)	<0.001
Abnormal WC vs. normal WC	1.01 (1.00, 1.01)	<0.001	1.01 (1.00, 1.01)	0.002	1.01 (1.01, 1.02)	<0.001	1.01 (1.01, 1.02)	<0.001
Diabetes duration (years), vs. <1year								
1–4	1.05 (0.93, 1.18)	0.466	1.00 (0.89, 1.11)	0.931	0.95 (0.85, 1.06)	0.383	1.06 (0.92, 1.21)	0.415
5–9	1.25 (1.11, 1.42)	<0.001	0.99 (0.88, 1.10)	0.813	1.99 (1.77, 2.22)	<0.001	1.69 (1.46, 1.95)	<0.001
≥10	1.11 (0.98, 1.26)	0.106	1.02 (0.91, 1.15)	0.682	1.44 (1.28, 1.61)	<0.001	1.45 (1.25, 1.68)	<0.001
Diabetes complications (yes vs. no)								
Cardiovascular	1.18 (1.08, 1.29)	<0.001	0.70(0.64, 0.77)	<0.001	0.93 (0.85, 1.01)	0.093	0.82 (0.73, 0.92)	<0.001
Cerebrovascular	1.37 (1.23, 1.52)	<0.001	0.93 (0.84, 1.02)	0.133	1.05 (0.95, 1.17)	0.350	1.15 (1.00, 1.34)	0.057
Peripheral	1.47 (1.14, 1.89)	0.003	0.96 (0.75, 1.23)	0.737	1.20 (0.92, 1.56)	0.178	1.53 (0.99, 2.37)	0.055
Nephropathy	1.68 (1.54, 1.84)	<0.001	0.96 (0.88, 1.04)	0.312	1.05 (0.96, 1.15)	0.258	1.40 (1.22, 1.60)	<0.001
Retinopathy	1.18 (1.08, 1.29)	<0.001	1.05 (0.96, 1.15)	0.258	0.96 (0.88, 1.05)	0.385	1.07 (0.94, 1.20)	0.305
Neuropathy	1.13 (1.03, 1.24)	0.009	1.06 (0.97 1.15)	0.211	1.47 (1.34, 1.61)	<0.001	1.29 (1.13, 1.47)	<0.001

## Discussion

Although the attainment of integrated glycemic, blood pressure and lipid goal is recommended by the current diabetes treatment guidelines, a smaller percentage of obese or overweight diabetes patients were able to achieve 3B goals compared with normal-weight patients. Furthermore, we observed that central obesity was significantly correlated with failure to achieve the 3B goal. To our knowledge, the present analysis is a first study to systematically assess the effect of obesity on 3B control in a large, geographically diverse sample of patients with type 2 diabetes. Given the upward trajectory in prevalence of obesity in diabetes, our findings clearly illustrate the importance of weight control for improving the 3B control rate and reducing the disease burden.

The health consequences of diabetes are compounded by overweight and obesity. In this study, 43.0% of diabetes patients were overweight and 16.7% were obese. In fact, we overestimated overweight and obesity based on the Chinese-specific BMI cutoffs of 24 and 28kg/m^2^ relative to the WHO criteria of 25 and 30kg/m^2^. Therefore, we recalculated the prevalence of overweight and obesity in this Chinese population using the WHO criteria, which gave prevalences of 38.77% (9503/24 512) for overweight and 7.14% (1750/24 512) for obesity in our study population. These rates are considerably lower than the percentages reported in a survey conducted in the United States in 1999–2002 [18]. In that survey, the combined prevalence of overweight and obesity (BMI ≥25kg/m^2^) was 85.2%, and the prevalence of obesity (BMI ≥30kg/m^2^) was 54.8%. A systematic literature review found a high variability in the prevalence of obesity in type 2 diabetes patients living in different countries [19]. Given the differences of ethnicities, country specified obesity criteria (e.g., BMI, WC, and waist/hip ratio) and obesity thresholds should be adapt for the country.

Hyperglycemia, hypertension, and dyslipidemia occur frequently in type 2 diabetes patients [19,20]. Iwahashi *et al*. reported that after oral glucose loading, insulin levels were higher in obese than in non-obese type 2 diabetes patients [21]. Obese (BMI ≥30kg/m^2^) type 2 diabetes patients treated at a clinic in the UK had higher blood pressure and triglyceride levels than nonobese patients, and that total cholesterol and glycemic control were worse in obese than nonobese male patients [22]. Similarly, our study found that overweight or obesity was associated with increased likelihood of comorbid hypertension and/or dyslipidemia. Overweight and obese patients also had higher SBP, DBP, fasting serum glucose, HbA1c, TC, LDL-C, and TG, but lower HDL-C levels than normal-weight patients. We also found that comorbidities occurred more often in overweight patients with central obesity than in those with a normal WC. Overall, these findings are in line with the Look AHEAD (Action for Health in Diabetes) study, a long-term trial of lifestyle intervention for weight loss that reported weight loss improved glycemic control, blood pressure, and lipid profiles in overweight type 2 diabetes patients [23].

In this study, the most frequently used oral antidiabetic agents were metformin (38.6%), sulfonylureas (28.9%), and α-glucosidase inhibitors (26.6%). A clinical trial conducted in Chinese patients with newly diagnosed type 2 diabetes reported no differences in the efficacy of metformin monotherapy to reduce HbA1c and LDL-C levels in normal weight, overweight, or obese patients after 16 weeks of treatment [24]. Another observational study reported similar reductions of glycaemia in patients with different BMIs after insulin detemir was added to treatment regimens that included oral glucose lowering drugs [25]. In this study, obese patients had higher average levels of TC, TG, and HbA1c, than other patients, but antihypertensive, lipid lowering and oral antidiabetic agents were used more frequently in obese than in other patients. This suggests that obese patients might require more complex pharmacological therapy to modify their cardiovascular risk factors.

Overall, only 14.3% of the enrolled patients achieved all 3B target goals. The proportion of 3B-controled patients decreased in overweight and obese patients, with only 10.1% of obese patients achieving all three therapeutic targets. Our findings confirm the negative impact of obesity on achieving 3B treatment goals, and that the negative effects were not offset by pharmacotherapy, emphasizing the known benefit of weight loss in diabetes patients [26,27]. The occurrence of hypertension, diabetes, and hyperlipidemia along with obesity has been referred to as a cardiometabolic risk factor cluster (CMRFC) [28]. More than 80% of the diabetes patients in this study had one or more concomitant cardiovascular risk factor, which made the choice of a treatment regimen complex and presented a significant challenge to effective achievement of 3B targets. The logistic regression analysis found that a BMI ≥24kg/m^2^ and an abnormal WC predicted failure to achieve the 3B therapeutic goals. Interestingly, our logistic regression results indicated that BMI seemed to be a stronger predictor than waist circumference. Determining this with certainty, however, will require long-term follow-up and further investigation.

Lifestyle can influence achievement of therapeutic goals for control of hyperglycemia, hypertension, and dyslipidemia in diabetic patients. In this study, alcohol consumption and a sedentary lifestyle were reported by more obese than normal weight patients. Frequent exercise might be of benefit in achieving 3B control. Qin *et al*. proposed that obesity and physical activity have an additive impact, and that prevention of either obesity or physical inactivity effectively reduces the risks of diabetes and its comorbid cardiometabolic diseases [29]. Physical activity may increase insulin sensitivity, glucose disposal, and oxidation of free fatty acids [29]. Moreover, the benefits of physical activity may extend beyond weight loss alone to include reduction of systemic and vascular inflammation [30]. Thus, 150 min/week of regular physical activity has been recommended as an intervention in diabetes treatment [10]. However, some studies have found that exercise had no measurable effect on glucose regulation in obese individuals without concomitant weight loss, and that the beneficial effect of exercise on metabolic complications seems only achievable when accompanied by weight loss [29]. In this study, smoking and drinking appeared to be independent risk factors for the failure to attain 3B goals. Therefore, overweight or obese patients with type 2 diabetes should be encouraged to adopt preventive lifestyle interventions, including frequent exercise and restriction of smoking and drinking habits.

This analysis of a large, geographically diverse population of type 2 diabetes patients adds to our knowledge of the negative effects of overweight or obesity on control of cardiometabolic risk in patients with type 2 diabetes. The main study limitation was its cross-sectional design, which made it difficult to confirm a causal relationship between obesity and failure to achieve treatment goals. This needs to be further explored in subsequent follow-up analyses of the 3B Study. Also, because the WC data was incomplete in nearly half the patients, the difference in 3B control achieved by centrally obese and non-centrally obese patients might be subject to selection bias. In addition, we did not include data on adherence to antidiabetic medication in the analysis. Finally, because dietary data were not available in the 3B Study, the relationships between diet and obesity or diet and 3B control could not be evaluated.

## Conclusions

Obesity was associated with poor control of blood glucose, blood pressure and blood lipids. The risk of cardiovascular complications thus remained increased in obese patients even though they were receiving more complex pharmacotherapy regimens than normal weight patients were. More attention and resources should be focused on encouraging and achieving weight loss and increased physical activity in patients with type 2 diabetes.

## Supporting Information

S1 Appendix Investigators List. (DOCX)
