# Supplementary material for: Prevalence of Obesity and Its Influence on Achievement of Cardiometabolic Therapeutic Goals in Chinese Type 2 Diabetes Patients: An Analysis of the Nationwide, Cross-Sectional 3B Study
Source: PLoS One. 2016 Jan 4;11(1):e0144179. doi: 10.1371/journal.pone.0144179 (PMC4699817; doi:10.1371/journal.pone.0144179)
Supplement: S1 Appendix — (DOCX) [file pone.0144179.s001.docx]

**S1 Appendix** Investigators List (by hospital)

1. Peking University People’s Hospital

*Zhun Sui, Mei Wang, Yingli Chen, Rongjing Ding, Dayi Hu, Linong Ji*

1. Beijing Hospital, Ministry of Health

*Lixin Guo, Qi Pan, Hua Wu*

1. Xuanwu Hospital Capital Medical University

*Huili Chen, Haixia Hou, Qi Hua, Yanling Wang*

1. Anzhen Hospital Capital Medical University

*Hong Chen, Xin Du, Changsheng Ma, Hong Tao*

1. Peking University First Hospital

*Yong Huo*

1. Peking Union Medical College Hospital

*Yuxiu Li, Zengyi Li, Xiaoping Xing*

1. China-Japan Friendship Hospital

*Yanyan Chen, Guangwei Li, Wenge Li, Xiaoxia Shen*

1. The General Hospital of the People's Liberation Army

*Nan Jin, Jing Li, Yiming Mu, Changyu Pan*

1. Beijing Shi Jing Shan Hospital

*Guogang Li, Weihua Li, Shanshan Lin, Changyang Su, Mingsheng Wang*

1. China Meitan General Hospital

*Hongmei Li, Zhenwei Shi*

1. Huguoshi Chinese Medicine Hospital Beijing University of Chinese Medicine

*Jian Huang, Bei Yang*

1. Daxing Chinese Medicine Hospital Beijing University of Chinese Medicine

*Li Ma, Jie Zhang*

1. Beijing Pinggu Hospital

*Yufeng Li, Cuiling Zhao*

1. Nanyuan Hospital

*Kaijie Yang, Jixia Zhao*

1. Shichahai Community Health Service of Xichen District Beijing

*Junqing Liu*

1. Beijing University of Posts and Telecommunications Community Health Service Center

*Xiaoyan Wang*

1. Puhuangyu Community Health Service Center of Beijing

*Yan Zhang*

1. Beijing Jiaotong University Hospital

*Manhong Li*

1. The Second Affiliated Hospital of Dalian Medical University

*Zhuo Li, Benli Su, Jiuyang Zhao, Lin Zhao*

1. Shenyang No. 4 People's Hospital

*Yanhe Cui, Jinsong Kuang, Man Li, Yinjun Li, Weiguang Luo*

1. Affiliated Zhongshan Hospital of Dalian University

*Shaokui Liu, Xiaomei Wang*

1. Second Affiliated Hospital of Jilin University

*Bin Liu, Yu Liu, Lijuan Wang*

1. Liaoning Power Center Hospital

*Mingyu Gao*

1. The Fourth Affiliated Hospital of Chinese Medicine University

*Zhiying Duan, Hongmei Ji, Yuanzhe Jin, Zongqian Wang*

1. Dalian Lvshunou People's Hospital

*Lu Chen, Danqi Wu, Shumin Yang*

1. Dalian Fifth People's Hospital

*Xingjia Liu, Mingrui Lv, Yili Ma, Li Wang, Rui Yu*

1. Diabetes Treatment Center Of Liaoning Province

*Bin Gao*

1. China–Japan Union Hospital and The First Affiliated Hospital of JILIN University

*Feng Liu, Qing Wang, Dongyan Xu, Ping Yang*

1. Changchun People's Hospital

*Pin Fu, Kaiping Mu*

1. Changchun Chaoyang District People's Hospital

*Ruijie Meng, Yu Zhao*

1. Dalian ShaHeKou XingHaiWan Garden Community Health Service Center

*Tinggui Gao, Yunshun Li*

1. Dalian ShaHeKou HeiShiJiao baiyun Community Health Service Center

*Shuqin Bao*

1. Shenyang HuangGuOu Longjiang Community Health Service Center

*Xia Cao*

1. Tiexiqu WeiGong Community Health Service Center

*Xianying Liu, Jianbo Zhao, Shenyang*

1. Xijing Hospital

*Qiuhe Ji*

1. Chinese PLA 323 Hospital

*Peijun Mao, Jixian Ye*

1. Chinese PLA 451 Hospital

*Dafang Wu, Yan Zhou*

1. The Second Hospital of Lanzhou University

*Jiangong Ren*

1. Gansu Provincial Hospital

*Jin Liu*

1. Xinjiao People’s Hospital of Haizhu District Guangzhou

*Zhaoqi He, Zhen Que*

1. Red Cross Hospital of Haizhu District Guangzhou

*Liping Li, Zhizhao Zhou*

1. Hulin Street Community Health Service Center of Liwan District Guangzhou

*Yi Lin, Xiangmin Xu*

1. Hongshan Street Community Health Service Center of Kaifu District Changsha

*Cuiwei Zheng*

1. West China School of Medicine Sichuan University

*Dawei Chen, Ping Fu, Xing Kang, Fang Liu, Xingwu Ran*

1. Second people's hospital of Chengdu

*Xiaoyun Chen, Bing Huang, Qiu Li, Xiance Luo*

1. First People's Hospital Of Chengdu

*Jie Gao, Zheng Li, Zhiming Lu, Yahui Yan*

1. Xinqiao Hospital of Third Military Medical University

*Zihui Xu, Houdi Zhou*

1. The First affiliated Hospital of Chongqing Medical University

*Han Lei, Qifu Li, Changhong Zhao*

1. The Second affiliated Hospital of Chongqing Medical University

*Yan Cheng, Changhui Guo, Shiguo Tang, Yanping Xu, Gangyi Yang,Yuehui Yang*

1. Hospital of Tradition Medicine LS.SC

*Kang Peng, Fenyuan Wu*

1. Hospital of Tradition Medicine MS.SC

*YangHong Wang*

1. Sixth People's Hospital Of Chengdu

*Yu Wang*

1. Fudan University Huashan Hospital

*Jing Chen, Chuanming Hao, Rengming Hu, Yun Li, WeiLing Qi, Haiming Shi, Chaoyun Zhang*

1. The First Affiliated Hospital of Shanghai JiaoTong University

*Yun Jiang, Shaowen Liu, Yongde Peng, Wei Wang, Weijie Yuan, Qing Yu*

1. Huadong Hospital Affiliated of Fudan University

*Lei Cai, Jiao Sun*

1. Shanghai Changzheng Hospital

*Ru Ding, Zhimin Liu, ChangLin Mei , Suxing Tuo, Zonggui Wu, Bei Zhang*

1. Tongji University affiliated Tenth People's Hospital

*Ai Peng, Shen Qu, Yaxiang Song, Yidong Wei, Yawei Xu, Peng Yang*

1. Jiangsu province hospital

*Tao Yang, Qingxin Yuan*

1. Shanghai Post and Telecommunications Hospital

*Yi Xin*

1. Shanghai Pudong New Area public Hospital

*Mingjun Gu, Meihua Guo, Lianyong Liu, Jun Lu, Jianping Qiu, XiaoLing Pi, Xumin Shen*

1. Shanghai Central Hospital of Shanghai Changning District

*Shan Huang, Xiaohong Jiang*

1. Central Hospital of Shanghai Minxing District

*Chengjun Chen, Xia Chen, Xudong Xu, Jialin Yang, Dadong Zhang, Yu Zhang*

1. Shanghai Luwan District Central Hospital

*Ling Chen, Yuwei Huang, Hui Li, Qiang Lu, Hong Shao, Ying Shen, Jianrong Zhao, Zhen Zhu*

1. Central Hospital of Shanghai Jingan District

*Jianrao Lu, Jun Wang, Xiu’e Zhao*

1. Central Hospital of Shanghai Songjiang District

*Xiujuan Zang, Lijuan Zhang*

1. Shanghai Yangpu District Yin hang Community Health Service Center

*Rong Li, Yumei Ma*

1. Shanghai Yangpu District Bridge Community Health Service Center

*Ming Cui, Yanhong Zhou*

1. Shanghai Jingan District community health service center in Jiangning

*Weifeng Chen,Tianhan Shen*

1. Shanghai Qingpu District Zhao Zhen Community Health Service Center

*Qing Gu, Meijuan Zhu*

1. Shanghai Minhang District Urban Community Health Service Center

*Hua Hang, Chunxiao He*

1. The Second People’s Hospital Haizhu District Guangzhou

*Yonglian Xie, Jixuan Zhao, Minling Zhao*

1. The Second People’s Hospital of Shanxi

*Wenli Liu, Lili Pa*

1. Liwan Hospital Guangzhou Medical University

*Hongbing Luo*

1. Workers' Hospital of Xi'an Aero-engine Group Co., Ltd.

*Yu’nan Jia*

1. Xi An Huxian Hospital

*Yun’an Wang*

1. Zhongshanmen Community Medical Service Center of Xinchen District of Xi’an

*Yipeng Hou, Bing Xia*

1. The Second Affiliated Hospital of Sun Yet-sun Hospital

*Guojuan Lao, Yan Li*

1. Xi An Hansengzhai Community Medical Service Center of Xinchen District

*Jiang Wu,*

1. Shangde Hospital of Xi’an

*Mingzhao Yang*

1. The Third Affiliated Hospital of Sun Yet-sun Hospital

*Ling Chen, Hongrong Deng, Ruimin Dong, Jin Li, Xun Liu, Tanqi Lou, Jianping Weng, Longyi Zeng*

1. Xi An Hujiamiao Hospital of Xinchen District

*Ji Li*

1. The First Affiliated Hospital of Sun Yet-sun Hospital

*Ailing Chen, Yanbing Li*

1. General Hospital of Guangzhou Military Command of PLA

*Aiming Deng, Jian Qiu, Junrong Tong, Lin Xu, Lili Ye, Xianyang Zhong*

1. Nanfang Hospital

*Ying Cao, Xiaojing Hu, Yaoming Xue*

1. Xiangya Hospital Central-South University

*Meng Jiang, Mingxiang Lei*

1. The Second Xangya Hospital Central-South University

*Weili Tang, Yu Zhou, Zhiguang Zhou*

1. Bingong 521 Hospital Xi’an

*Xudan Zhen*

1. Dongfeng Branch of the Second People’s Hospital Yuexiu District Guangzhou

*Xiangwei Chen*

1. Guangzhou Diabetes Hospital

*Maolin Luo*

1. Finance & Trade Hospital of Hunan Province

*Xiao Xiong, Liping Zhu*

1. Xi An Huashan Centre Hospital

*Qinli Fan*

1. First People's Hospital Of Longquanyi District Of Chengdu

*Lihua Liu, Li Zhong*

1. People's Hospital of Pi County

*Tianhu Liu, Yumei Zhang*

1. Chongqing Tung Wah Hospital

*Jinghui Lu*

1. Wuhou Community Health Service Center of Wuhou District of Chengdu

*Lin Yang, Wenyi Yang*

1. West City Community Health Service Center of Xindu District of Chengdu

*Liuli He, Xiaohua Wu*

1. Yuling Community Health Service Center of Wuhou District of Chengdu

*Xilian Gao, Jing Jiang*

1. Southwest Computer Company Worker's Hospital of Chongqing

*Yana Xu, Bo Zhang*

1. Yuxi Hospital of Chongqing

*Rongping Tian, Xinjian Zhou*

1. General Hospital Of Chongqing Iron And Steel

*Xiaochun Teng*

1. Sichuan Academy of Medical Sciences & Sichuan Provincial People’s Hospital

*Mingjing Bao, Pengqiu Li, Limei Li, Yichuan Wu, Yang Xuan, Yan Yang, Xuejun Zhang*

1. Third people's hospital Chengdu

*Hui He, Jingyu Liu, Li Zhang*

1. Second People's Hospital Of Jiulongpo District Of Chongqing

*Qiu Xu, Hongju Zhong*
